# Inhibition of CD44 intracellular domain production suppresses bovine articular chondrocyte de-differentiation induced by excessive mechanical stress loading

**DOI:** 10.1038/s41598-019-50166-4

**Published:** 2019-10-17

**Authors:** Yasumori Sobue, Nobunori Takahashi, Yoshifumi Ohashi, Mochihito Suzuki, Tsuyoshi Nishiume, Tomonori Kobayakawa, Kenya Terabe, Warren Knudson, Cheryl Knudson, Naoki Ishiguro, Toshihisa Kojima

**Affiliations:** 1 Nagoya University Graduate School of Medicine, Department of Orthopedic Surgery, 65 Tsurumai, Showa, Nagoya 466-8550 Japan; 2 Kobayakawa Clinic, Department of Orthopedic Surgery, 1969 Kuno, Fukuroi, 437-0061 Japan; 3 The Brody School of Medicine, East Carolina University, 600 Moye Boulevard, Greenville, NC 27834-4354 North Carolina USA

**Keywords:** Biochemistry, Chemical biology

## Abstract

CD44 fragmentation is enhanced in chondrocytes of osteoarthritis (OA) patients. We hypothesized that mechanical stress-induced enhancement of CD44-intracellular domain (CD44-ICD) production plays an important role in the de-differentiation of chondrocytes and OA. This study aimed to assess the relationship between CD44-ICD and chondrocyte gene expression. Monolayer cultured primary bovine articular chondrocytes (BACs) were subjected to cyclic tensile strain (CTS) loading. ADAM10 inhibitor (GI254023X) and γ-secretase inhibitor (DAPT) were used to inhibit CD44 cleavage. In overexpression experiments, BACs were electroporated with a plasmid encoding CD44-ICD. CTS loading increased the expression of ADAM10 and subsequent CD44 cleavage, while decreasing the expression of SOX9, aggrecan, and type 2 collagen (COL2). Overexpression of CD44-ICD also resulted in decreased expression of these chondrocyte genes. Both GI254023X and DAPT reduced the production of CD44-ICD upon CTS loading, and significantly rescued the reduction of SOX9 expression by CTS loading. Chemical inhibition of CD44-ICD production also rescued aggrecan and COL2 expression following CTS loading. Our findings suggest that CD44-ICD is closely associated with the de-differentiation of chondrocytes. Excessive mechanical stress loading promoted the de-differentiation of BACs by enhancing CD44 cleavage and CD44-ICD production. Suppression of CD44 cleavage has potential as a novel treatment strategy for OA.

## Introduction

Osteoarthritis (OA) is characterized by both the degradation of articular cartilage and the destruction of joints as a result of loss of homeostasis in articular cartilage^1^. Articular chondrocytes in OA patients undergo de-differentiation, resulting in a decreased amount of synthesized cartilage matrix^2^. De-differentiation of chondrocytes is accompanied by a reduction in expression of SOX9, aggrecan, and type 2 collagen, and induction of a fibroblastic phenotype characterized by the expression of type 1 collagen^3–5^. OA is caused by multiple factors such as genetics, aging, obesity, and mechanical stress. Depending on its intensity, mechanical stress loading can promote either catabolism or anabolism^6^. Excessive mechanical stress loading, however, can induce chondrocyte de-differentiation, articular cartilage degradation, and OA onset^7–9^, and alter the expression of various catabolic and anabolic genes that regulate cartilage remodeling and turnover, potentially leading to proteolytic cleavage of the extracellular matrix^10^. However, the molecular mechanism underlying the influence of excessive mechanical stress loading on OA changes is not yet fully understood.

CD44 is a single-pass transmembrane receptor that serves as the primary receptor for hyaluronan (HA). Interactions between CD44 and HA are important for maintaining proteoglycan-rich pericellular matrices and cartilage homeostasis^11^. Disruption of the interaction between CD44 and HA can impact matrix metabolism and repair via CD44-related intracellular signaling transduction in chondrocytes^12,13^. Previous studies have reported that CD44 is proteolytically cleaved in a number of tumor cell types and chondrocytes in OA patients^11,14^. CD44 cleavage involves the proteolytic cleavage of the extracellular domain of CD44 by a metalloproteinase (MT1-MMP, ADAM17, or ADAM10)^15^. We previously reported that ADAM10 was the primary metalloproteinase of this first-step cleavage of CD44 in bovine articular chondrocytes (BACs)^16^. The metalloproteinase releases a 70 kD CD44 ecto-domain into the extracellular matrix, leaving a 18–20 kD C-terminal truncation fragment within the plasma membrane (termed CD44-EXT). The CD44-EXT fragment is then cleaved within the intramembrane domain by γ-secretase, releasing a 15 kD intracellular domain (CD44-ICD) into the cytoplasm^11^.

Transient receptor potential vanilloid 4 (TRPV4), a Ca^2+^-permeable osmo-mechano-TRP channel^17^, was recently reported to act as mechanoreceptor and mediator of chondrogenic differentiation in porcine articular chondrocytes^18^. We previously found that mechanical stress loading increased ADAM10 expression and CD44 cleavage via TRPV4 activation in a human chondrocyte cell line (HCS)^19^. The release of these CD44 fragments can negatively impact chondrocyte function. For example, the release of CD44-ICD into the cytoplasm of chondrocytes has been reported to competitively block interactions between full-length CD44 and cytoskeletal adaptor proteins. These interactions are required to stabilize the retention of pericellular matrix in chondrocytes^20^.

Here, we hypothesized that CD44 cleavage and subsequent CD44-ICD production in chondrocytes have negative effects on the maintenance of chondrocyte differentiation under conditions of excessive mechanical stress loading. Accordingly, this study aimed to assess the effect of CD44-ICD on the de-differentiation of primary BACs induced by excessive mechanical stress loading.

## Results

### Mechanical stress loading

**Figure 1:**
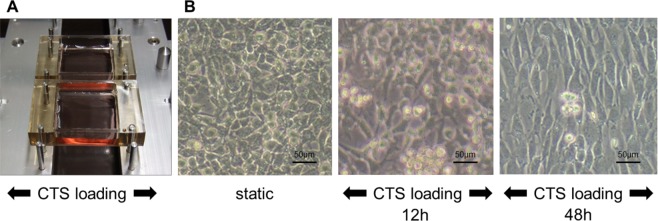
Morphological changes in bovine articular chondrocytes (BAC) subjected to cyclic tensile strain (CTS) loading. (**A**) Isolated BAC monolayers cultured on specialized silicone chambers were stimulated by CTS loading using STB-140 (STREX Inc.). (**B**) (left panel) Static monolayer BACs had a cobblestone-like appearance. (middle panel) After 12 hours of CTS loading under conditions of 0.5 Hz and 20% elongation. (right panel) After 48 hours of CTS loading under conditions of 0.5 Hz and 20% elongation, BACs became spindle-shaped with the long axis orthogonally oriented to the axis of CTS loading.

An automated cell stretching system (STB-140; STREX, Japan) stretches silicon chambers, and adherent cells are stretched perpendicular to the direction of mechanical stress loading (Fig. 1A). Upon stretching, BACs became spindle-shaped at 48 hours, with the long axis orthogonally oriented to the axis of stress loading (Fig. 1B). However, upon 12 hours of CTS loading, we did not observe apparent morphologic change.

### Mechanical stress loading increases ADAM expression

**Figure 2:**
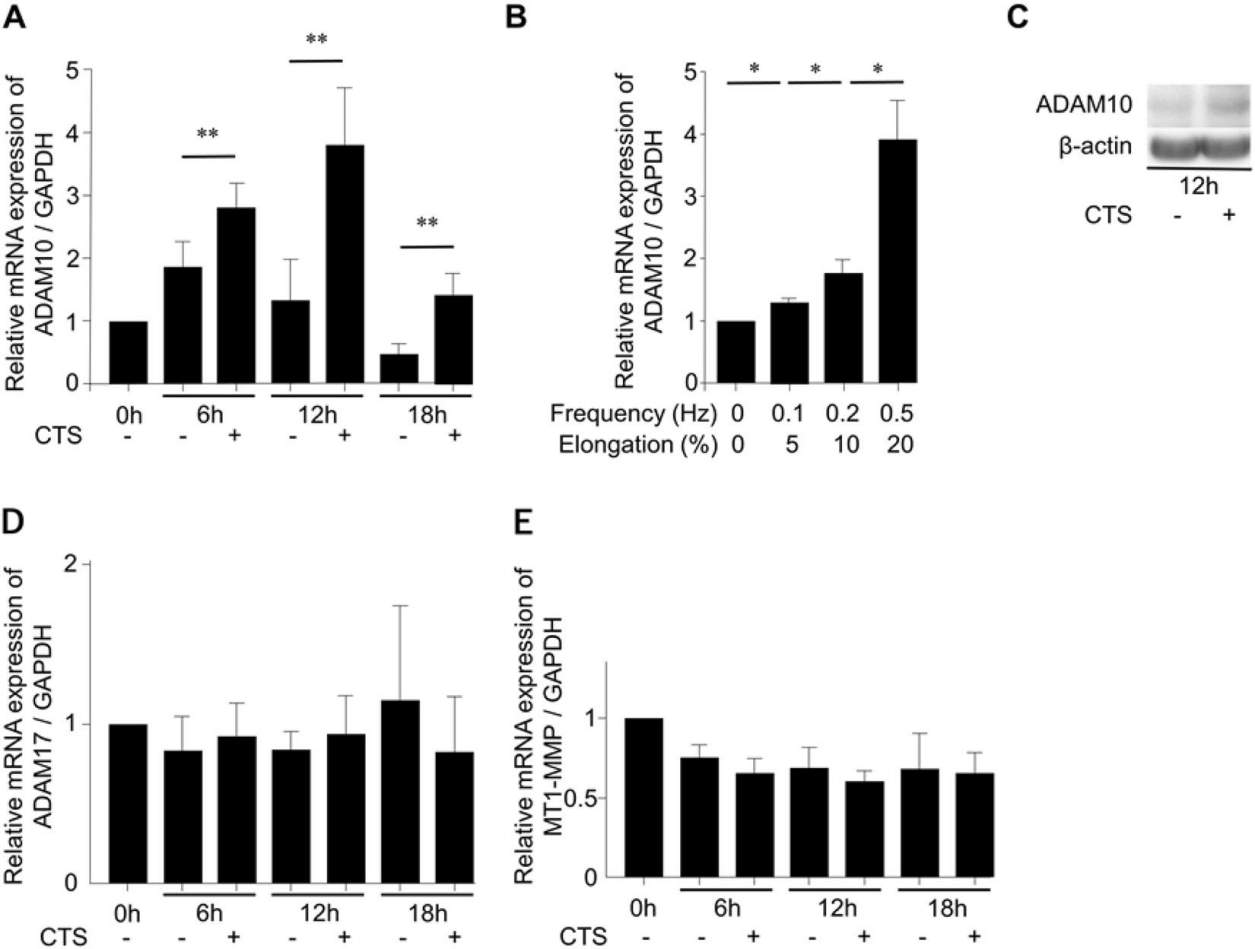
Increased expression of disintegrin and metalloproteinase domain-containing protein 10 (ADAM10) by cyclic tensile strain (CTS) loading. (**A**) Expression of ADAM10 mRNA was significantly increased by CTS loading under conditions of 0.5 Hz and 20% elongation at various time points (six independent experiments). (**B**) Intensity-dependent increase of ADAM10 mRNA expression induced by CTS loading for 12 hours. (**C**) Western blot analysis revealed increased ADAM10 protein expression induced by CTS loading under conditions of 0.5 Hz and 20% elongation for 12 hours. (**D**,**E**) There was no significant increase in ADAM17 or MT1-MMP mRNA expression by CTS loading under conditions of 0.5 Hz and 20% elongation. Values are mean ± standard deviation from six independent experiments. **p < 0.01, *p < 0.05.

ADAM10 expression increased with mechanical stress loading in a time and intensity- dependent manner. ADAM10 mRNA expression levels significantly increased gradually after mechanical stress loading (frequency, 0.5 Hz; elongation, 20%) started and reached most apparent levels at 12 hours (Fig. 2A). In a comparison of different strengths, the highest level of ADAM10 mRNA expression was observed under conditions of 0.5 Hz and 20% elongation at 12 hours (Fig. 2B). Consistent with mRNA expression, Western blot analysis confirmed that ADAM10 protein expression increased under the same conditions as well (Fig. 2C, Sup. 1). Subsequent mechanical stress loading experiments used these conditions (i.e., 0.5 Hz and 20% elongation), unless otherwise noted.

No significant changes were observed in the expression of ADAM17 and MT1-MMP mRNA (Fig. 2D,E). These results suggest that, consistent with a previous report using HCS^19^, mechanical stress loading increases ADAM10 mRNA expression.

### Mechanical stress loading induces CD44 cleavage and chondrocyte de-differentiation

**Figure 3:**
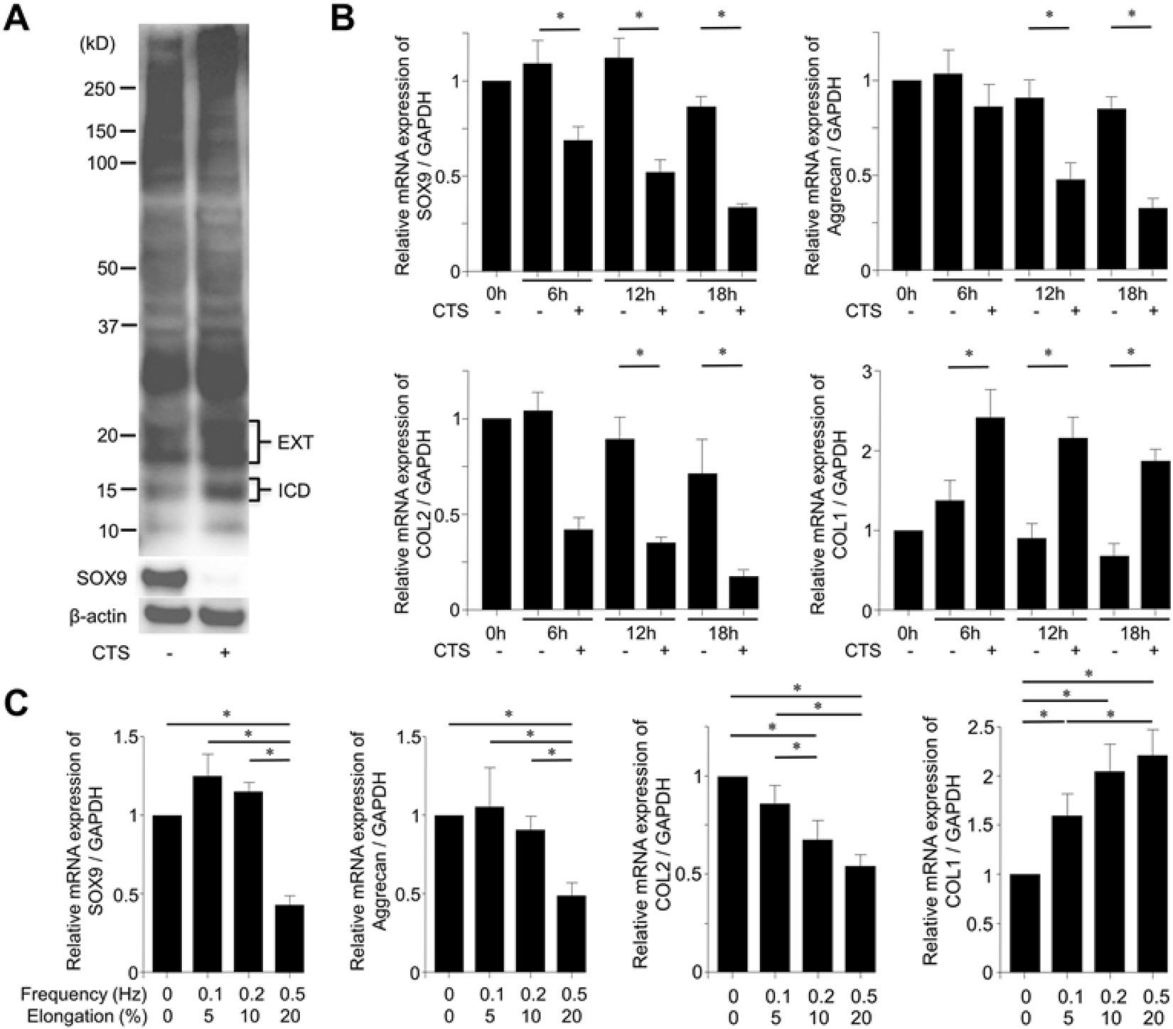
Cyclic tensile strain (CTS) loading induces CD44 cleavage and de-differentiation of bovine articular chondrocytes (BACs). (**A**) Western blot analysis showing enhanced CD44 cleavage and decreased SOX9 expression induced by CTS loading (0.5 Hz and 20% elongation) for 12 hours. Enhanced CD44 cleavage was noted as 18–20 kD doublet C-terminal fragment (CD44-EXT) bands and the 15 kD intracellular domain (CD44-ICD) band by the polyclonal anti-CD44 antibodies. (**B**) Changes in articular chondrocyte-related genes by CTS loading. CTS loading significantly decreased the mRNA expression of chondrocyte differentiation markers (SOX9, aggrecan, and collagen type 2 [COL2]). In contrast, mRNA expression of a chondrocyte de-differentiation marker (collagen type 1 [COL1]) was significantly increased by CTS loading. (**C**) CTS loading decreased the mRNA expression of SOX9, aggrecan, and COL2 mRNA, and increased the expression of COL1 mRNA, at 12 hours. Values are mean ± standard deviation from six independent experiments. *p < 0.05.

We next examined the relationship between mechanical stress loading and CD44 cleavage and changes in the expression of genes related to chondrocyte differentiation. Western blot analysis revealed an increase in CD44-ICD (approximately 15 kD) and CD44-EXT (18–20 kD) bands following mechanical stress loading. Interestingly, mechanical stress loading also decreased SOX9 protein expression (Fig. 3A, Sup. 2). Consistent with this, mechanical stress loading significantly decreased the mRNA expression of SOX9 at 6, 12, and 18 hours, AGC at 12 and 18 hours, and COL2 at 12 and 18 hours. In contrast, COL1 mRNA expression was significantly increased at 6, 12, and 18 hours (Fig. 3B). Similar to the effect on ADAM10 expression, mechanical stress loading (0.5 Hz and 20% elongation) showed the strongest effect on the expression of chondrocyte differentiation-related genes at 12 hours (Fig. 3C).

### Induction of chondrocyte de-differentiation by CD44-ICD overexpression

**Figure 4:**
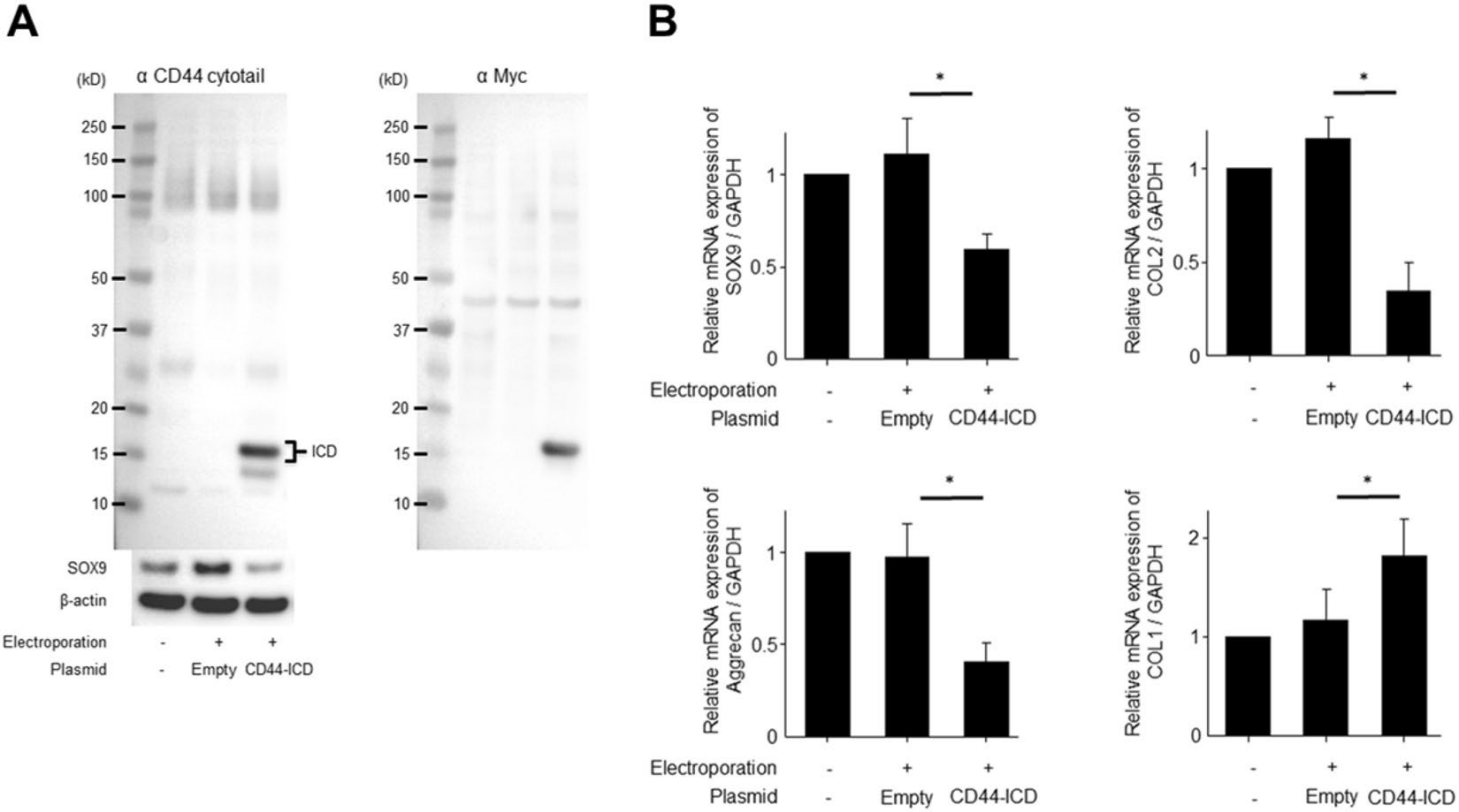
Effect of CD44-ICD overexpression on bovine articular chondrocytes (BACs) de-differentiation. (**A**) BACs were transfected with plasmid DNA expressing CD44-ICD (pCMV/myc/cyto-CD44ICD) or empty plasmid as a control using an electroporator (NEPA21). A strong band of about 15 kD was observed in BACs transfected with the CD44-ICD plasmid, which was detected when probed with both anti-CD44 cytotail and anti-Myc antibodies, and SOX9 levels were reduced in these cells. (**B**) CD44-ICD overexpression significantly decreased the mRNA expression of chondrocyte differentiation markers (SOX9, aggrecan, and collagen type 2 [COL2]). In contrast, CD44-ICD overexpression significantly increased the mRNA expression of a chondrocyte de-differentiation marker (collagen type 1 [COL1]). Values are mean ± standard deviation from six independent experiments. *p < 0.05.

The effect of CD44-ICD overexpression on the expression of chondrogenic differentiation-related genes was also assessed. As shown in Fig. 4A, two CD44-ICD bands of about 15 kD (a strong band and weak band) were observed in lysates of BACs transfected with the CD44-ICD plasmid by Western blot, while corresponding bands were absent in BACs that were not transfected or were transfected with control plasmid. The molecular weight of the weaker band is consistent with that of the natural form of CD44-ICD^11^. The molecular weight of the strong band is consistent with what would be expected from the addition of the myc-tag (approximately 1.2 kD), which was proven with the addition of anti-Myc antibody. SOX9 protein expression was reduced in BACs transfected with CD44-ICD, but not in controls (Fig. 4A).

CD44-ICD significantly decreased the mRNA expression of SOX9, aggrecan, and COL2, while increasing the expression of COL1 mRNA (Fig. 4B). This suggests that the ability to maintain the chondrocyte phenotype was disrupted by CD44-ICD overexpression. These results suggest that CD44-ICD overexpression or CD44-ICD production upon excess mechanical stress loading can promote the de-differentiation of articular chondrocytes.

### Rescue of SOX9 expression by chemical inhibitors of CD44-ICD production upon mechanical stress loading

**Figure 5:**
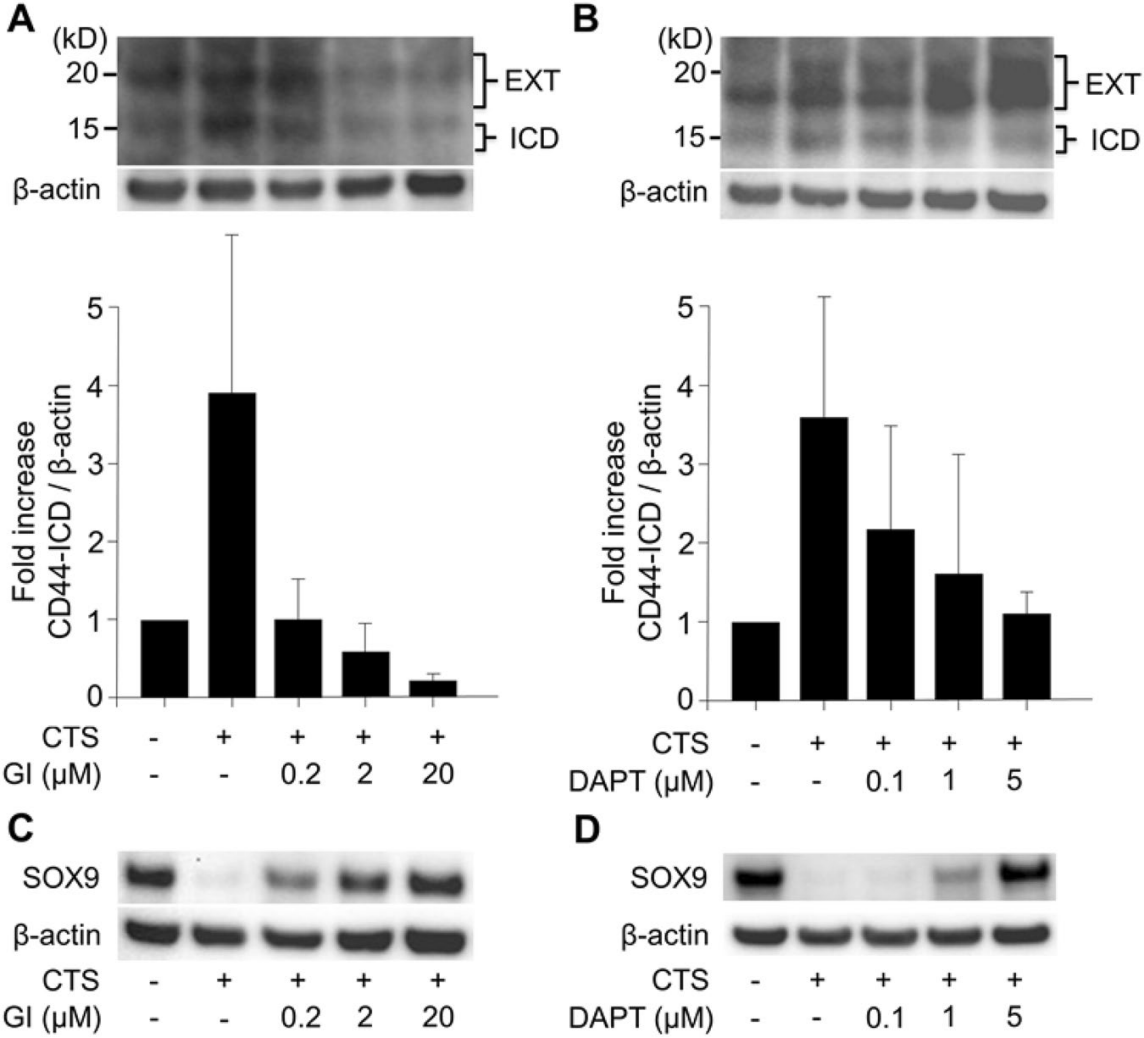
Effect of chemical inhibitors of CD44-ICD production on the reduced expression of SOX9 protein by cyclic tensile strain (CTS) loading. (**A**) GI254023X (GI), a chemical inhibitor of ADAM10, dose-dependently decreased both CD44-EXT and CD44-ICD bands upon CTS loading (0.5 Hz and 20% elongation for 12 hours). Densitometry of CD44-ICD/β-actin. Values are mean ± standard deviation from three independent experiments and expressed as n-fold increase compared to untreated control. (**B**) DAPT, a chemical inhibitor of γ-secretase, dose-dependently decreased production of the CD44-ICD band by CTS loading. At the same time, CD44-EXT accumulated in a dose-dependent manner. Densitometry of CD44-ICD/β-actin. Values are mean ± standard deviation of three independent experiments. (**C**,**D**) Both GI and DAPT effectively rescued the decrease in SOX9 expression by CTS loading (0.5 Hz and 20% elongation for 12 hours) in a dose-dependent manner.

To determine whether the decrease in SOX9 expression upon mechanical stress loading could be rescued by preventing CD44-ICD production, we subjected cells to mechanical stress loading in the presence of chemical inhibitors of CD44-ICD production. Pre-treatment of cells with GI254023X, an inhibitor of ADAM10 (the protease involved in the first step of CD44-ICD production), suppressed the production of both CD44-EXT and CD44-ICD upon mechanical stress loading in a dose-dependent manner (Fig. 5A). In contrast, pre-treatment of cells with DAPT, an inhibitor of γ-secretase (the protease involved in the second step of CD44-ICD production), significantly suppressed CD44-ICD production in a dose-dependent manner, but resulted in the accumulation of CD44-EXT (Fig. 5B). Both GI254023X and DAPT prevented the reduction in SOX9 protein expression in a dose-dependent manner. At concentrations of 20 µM for GI254023X and 5 µM for DAPT, the intensity of the SOX9 band remained similar to that in samples that were not treated with mechanical stress loading (Fig. 5C,D).

### Rescue of chondrocyte differentiation-related gene expression by chemical inhibitors of CD44-ICD production upon mechanical stress loading

**Figure 6:**
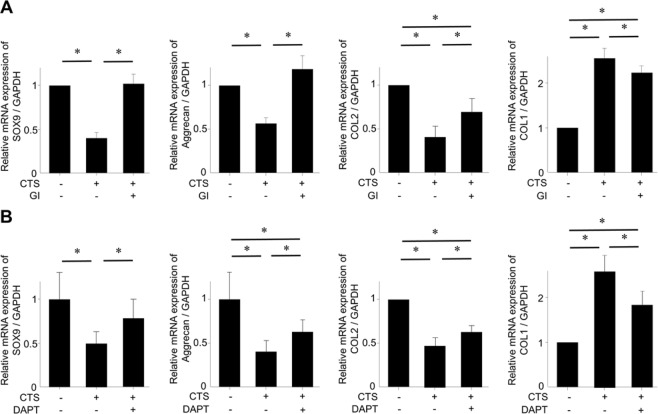
Effect of chemical inhibitors of CD44-ICD production on the expression of chondrocyte phenotype-related genes affected by cyclic tensile strain (CTS) loading. (**A**,**B**) Both GI254023X [GI] and DAPT significantly reversed the suppression of mRNA expression of SOX9, aggrecan, collagen type 2 (COL2) by CTS loading (0.5 Hz and 20% elongation for 12 hours). Moreover, the increase in mRNA expression of collagen type 1 (COL1) by CTS loading was significantly inhibited by GI and DAPT. Values are mean ± standard deviation from six independent experiments. *p < 0.05.

Given the ability of GI254023X and DAPT to rescue SOX9 protein expression, we next tested whether these inhibitors could prevent chondrocyte de-differentiation upon mechanical stress loading, as assessed by the expression of chondrocyte differentiation-related genes. Consistent with the results seen with SOX9 protein expression, both GI254023X (20 µM) and DAPT (5 µM) prevented the decrease in SOX9 mRNA expression upon mechanical stress loading (Fig. 6A,B). Similarly, these inhibitors also worked in the direction to prevent the decrease in mRNA expression of aggrecan and COL2. Conversely, the two inhibitors worked in the direction to prevent the increase in COL1 mRNA expression upon mechanical stress loading.

The effects of GI254023X and DAPT on preventing chondrocyte de-differentiation upon mechanical stress loading (as assessed by the expression of chondrocyte differentiation-related genes) were significant, but not as robust for some genes. GI254023X did not fully rescue mRNA levels of COL2 and COL1, and DAPT did not rescue mRNA levels of aggrecan, COL2, and COL1. In these gees, there were still significant differences between untreated control samples and those treated with GI254023X or DAPT. These results suggest that other factors might be involved in the chondrocyte de-differentiation process induced by excessive mechanical stress loading.

## Discussion

In this study, we found that excessive mechanical stress loading induces the de-differentiation of articular chondrocytes via CD44 cleavage and subsequent CD44-ICD production. Excessive mechanical stress loading significantly decreased the expression of genes involved in chondrocyte differentiation, such as SOX9. To our knowledge, this is the first report demonstrating the involvement of CD44-ICD in the de-differentiation of chondrocytes after mechanical stress loading. Our findings point to a potential strategy for treating OA that involves the suppression of CD44 cleavage.

We previously reported that proteolytic cleavage of CD44 was enhanced in articular cartilage derived from OA patients^11^ and that overexpression of CD44-ICD resulted in the loss of pericellular matrix in bovine articular chondrocytes^20^. However, there have been no reports on whether CD44-ICD itself plays a role in chondrocytes. In some tumor cells, CD44-ICD has been reported to function as a signaling molecule that translocates into the nucleus and activates transcription^15^. In the present study, we found that CD44-ICD altered the gene expression profile of chondrocytes. For instance, inhibiting CD44 cleavage using GI254023X or DAPT prevented changes in SOX9 expression by excessive mechanical stress loading, and overexpressing CD44-ICD decreased SOX9 expression in the chondrocytes.

The bovine articular chondrocytes were subjected to CTS loading on silicone chambers. Since we could not extract total protein samples directly from the chambers, the cells were trypsinized and centrifuged first. Trypsin-EDTA can cleave CD44 as well. Compared to the native CD44 cleavage pattern^11^, we can observe the diminished full length CD44 bands at ~85kD and the newly appeared strong bands at ~25kD (trypsin generated CD44-EXT) and some other faint bands in Fig. 3A. We cannot evaluate the CD44 fragmentation at 25–85kD when using the trypsinized cell samples. Importantly, the trypsin treatment does not affect the generation of native CD44-EXT (ADAM10 generated at ~20kD) and CD44-ICD. We can study native CD44 fragmentation status using the cells with trypsin treatment first.

In our previous study, simvastatin, a statin and therapeutic agent used to treat hypercholesterolemia, inhibited CD44 cleavage in bovine chondrocytes^16^. Inhibition of CD44 cleavage by simvastatin also resulted in improved retention of pericellular matrix. This protective effect was reversed by the addition of cholesterol and farnesylpyrophosphate. Based on this, we concluded that simvastatin exerts positive effects on chondrocytes by reducing CD44 cleavage and enhancing pericellular matrix retention. Statins have been reported to reduce cartilage degradation in a rabbit model of OA^21^. Thus, we speculate that supplementation of statins could have a chondroprotective effect through suppression of CD44-ICD production.

The effects of GI254023X and DAPT on changes in gene expression induced by mechanical stress loading slightly differed. Specifically, while GI254023X completely rescued the decrease in aggrecan expression to baseline levels, DAPT only partially, albeit significantly, rescued aggrecan expression. DAPT is a γ-secretase inhibitor which targets only the second step of CD44 cleavage into CD44-ICD. In contrast, GI254023X is a specific ADAM10 inhibitor which can inhibit all steps of CD44 cleavage by targeting the first step of cleavage. Thus, GI254023X inhibits both production of the CD44 ecto-domain and CD44-ICD. Since the CD44 ecto-domain can function as a decoy receptor for pericellular HA^22,23^, inhibiting its production could contribute to maintaining pericellular matrix homeostasis. In this regard, a detailed study regarding the function of the CD44 ecto-domain would be informative. Nonetheless, chemical inhibition of the CD44 cleavage pathway at two different steps led to retention of the chondrocyte phenotype under excessive mechanical stress loading.

Mechanical stress loading can promote either catabolism or anabolism depending on its intensity^6,24–26^. Lin *et al*. reported that excessive stress loading at 23% elongation and 0.5 Hz (which is similar to our present conditions) reduced SOX9 expression^27^. Since we studied the catabolic effects of mechanical stress loading, we applied conditions found to have the greatest effect on inducing the expression of ADAM10 (0.5 Hz and 20% elongation). We previously confirmed that similar conditions (1 Hz and 20% elongation) induced the strongest CD44 cleavage using the human chondrocyte cell line HCS 2/8^19^. However, this condition resulted in cell death of BACs in the present study.

ADAM10 functions as a membrane-anchored metalloproteinase and exists abundantly in degenerated and OA cartilage^28^. We previously reported that ADAM10 expression and CD44 cleavage were enhanced by mechanical stress loading in a human chondrocyte cell line^19^. The results from that study were confirmed using primary cells in the present study. CD44 cleavage itself may be considered a phenomenon that reflects cartilage degradation or OA^11^, but not necessarily the degeneration or de-differentiation of articular chondrocytes.

SOX9 is a transcription factor that acts as a determinative switch in chondrogenesis^29^. Since SOX9 regulates the expression of genes related to cartilage metabolism, it is important to demonstrate changes in its expression, along with changes in the expression of other genes involved in differentiation, when evaluating the loss of the chondrocyte phenotype^6,30,31^. In the present study, we demonstrated that excessive mechanical stress loading induced the de-differentiation of BACs, and that enhanced production of CD44-ICD by mechanical stress played an important role in the de-differentiation process.

The present study has some limitations. First, mechanical stress was applied using only one method. Other methods of mechanical stress loading exist, such as compression stress and shear stress. However, stretching stress may reflect a physiological reaction given a report that cells are always being stimulated when they are in an extended state^32^. Second, BACs were cultured on a two-dimensional surface. Thus, cell behavior may differ when cells are cultured in a three three-dimensional environment^33^. The monolayer-cultured chondrocytes used in this study may have been already in the process of de-differentiation before any mechanical stimulus was applied. Since there is evidence that de-differentiated chondrocytes have increased stiffness by strengthening membrane-actin adhesion^34^, we have possibility that the cells we used had lowered responsiveness to the mechanical stimulus. Three-dimensional cultured chondrocytes could adequately respond to mechanical stress loading and demonstrate more accurate data. Future studies that use three-dimensional cultures or animal models to confirm our present results are warranted.

In conclusion, we demonstrated that excessive mechanical stress loading increased ADAM10 expression and enhanced CD44 cleavage in primary BACs. Mechanical stress loading also significantly induced the de-differentiation of chondrocytes, with CD44-ICD playing an important role in this process. Thus, suppression of CD44 cleavage may serve as a therapeutic strategy for OA.

## Method

### Cell culture

BACs were isolated from full thickness slices of the articular surface of metacarpophalangeal joints of young adult steers (aged 18–24 months), which were provided by Nagoya City Central Wholesale Market. These slices were digested in 0.2% (0.05 g) Pronase (catalogue number: 537088, activity: ≥70,000 proteolytic units/g dry weight, Merck, Germany) for 1 hour at 37 °C and subsequently in 0.025% (0.00625 g) collagenase P (catalogue number: 11213865001, activity: >1.5 U/mg lyophilizate, Roche, Germany) overnight at 37 °C^11^. Cells were cultured in DMEM/Ham’s F12 medium with 1 × insulin-transferrin-sodium selenite (ITS), 4% FBS, 100 units/ml penicillin, 100 µg/ml streptomycin, and 0.25 µg/ml amphotericin at 37 °C in a 5% CO_2_ environment. The presence of ITS maintains the chondrocyte phenotype^30,35^. For mechanical stress loading experiments, 1 × 10^6^ cells were cultured on 10 cm^2^ dedicated silicone chambers (STB-CH-10, STREX, Japan), which were coated with type 1 collagen (CELLMATRIX, Nitta Gelatin, Japan). After static incubation for 48 hours in 4% FBS, cells were cultured in serum-free medium for 24 hours. Subsequently, cells were stimulated using the automated cell stretching system (STB-140; STREX, Japan) under serum-free conditions^19^. In certain experiments, cells were treated with drugs in serum-free medium 24 hours before mechanical stress loading^19^.

### Plasmid electroporation

A total of 1 × 10^6^ cells were mixed with a plasmid (10 µg) encoding CD44-ICD (pCMV/myc/cyto-CD44ICD), with the corresponding empty plasmid (10 µg) as the negative control, in Opti-MEM with serum- and antibiotic-free medium. The plasmid was constructed at East Carolina University as described previously^11^. Briefly, the human CD44-ICD coding sequence was subcloned into a pCMV/myc/cyto plasmid (pShooter, Invitrogen). PCR primers used for subcloning were designed to amplify the sequence corresponding to CD44-ICD (CD44 Ala^288^ to the stop codon following Val^361^) of full length human CD44H. Primer sequences were as follows: 5′-GTCGACGCAGTCAACAGTCGAAGAAGGTGTGG-3′ (containing a Sal I restriction site) and 5′-TTACACCCCAATCTTCATGTCCACATTC-3′^11,20^. This pShooter plasmid provides high efficiency delivery of the transgene product (CD44-ICD) directly into the cytoplasm.

The pCMV/myc/cyto-CD44ICD plasmid (or corresponding control plasmid) and cells were added to 2-mm gap cuvettes and stimulated by a NEPA21 electroporator (NEPAGENE, Chiba, Japan) under optimized conditions (voltage: 275 V, pulse width: 2.5 ms, gene transfer efficiency: 80%, survival rate: 85%)^36^. Cells were evaluated 24 hours after electroporation. Consistent with its expected molecular weight, CD44-ICD migrated to a position higher than 15 kD in Western blot analysis due to the myc tag.

### Chemical treatments

Previous studies have reported that CD44 is proteolytically cleaved to produce three fragments^11,37^. Cleavage of CD44 mediated by the first metalloprotease (ADAM 10) generates extracellular fragments and a C-terminal fragment (CD44-EXT). CD44-EXT is subsequently cleaved by γ-secretase to produce the CD44 intracellular domain (CD44-ICD). ADAM10 inhibitors can block all steps of CD44 cleavage, and γ-secretase inhibitors can prevent the second step of cleavage, i.e., the formation of CD44-ICD^11,16^. In experiments testing the effects of inhibiting these proteases, cells were treated with GI254023X (TOCRIS bioscience, UK) or N-[N-(3,5-diflurophenylacetate)-L-alanyl]-(S)-phenylglycine t-butyl ester (Calbiochem, USA) (DAPT), which are pharmacological inhibitors of ADAM10 and γ-secretase, respectively. GI254023X and DAPT were resuspended in DMSO at concentrations described in previous reports^16,38^.

### Real-time RT-PCR

Total RNA was extracted with the RNeasy Mini Kit (Qiagen, Germany). Reverse transcription was performed using the High Capacity cDNA Reverse Transcription Kit (Applied Biosystems, USA). Real time RT-PCR was carried out using a Light cycler System with FastStart Master SYBR Green PLUS (Roche, USA). Primers for ADAM10, ADAM17, MT1-MMP, CD44, SOX9, type2 collagen (COL2A1 [COL2]), type1 collagen (COL1A2 [COL1]), aggrecan, and GAPDH were synthesized by Sigma-Aldrich (USA). The following primers were used: ADAM10, forward primer 5′-CATCTGGGGACAAACTTAACAACA-3′, reverse primer 5′-CCCATTTCCACAAATAGGTTGGC-3′; ADAM17, forward primer 5′-TGCAAAGGCGTGTCTTATTGTAC-3′, reverse primer 5′-GCACAGGACTCCAGCCTCTG-3′; MT1-MMP, forward primer 5′-CCGTCCCCGATAAGCCCAAA-3′, reverse primer 5′-CCAGAACCAACGCTCCTTGAAG-3′; CD44, forward primer 5′-TCTGCAAGGCCTTTAATAGCACGC-3′, reverse primer 5′-GTTCGCAGCACAGATGGAATTGG-3′; SOX9, forward primer 5′-CATGAAGATGACCGACGAGCAG-3′, reverse primer 5′-GGGGAACGTGTTCTCCTGGG-3′; COL2A1, forward primer 5′-GTGGATTTGATGAGAAGGCTGGT-3′, reverse primer 5′-CCTTGAGGTCCGGGAGCAC-3′; COL1A2, forward primer 5′-GCATTAGGGGTCACAATGGTCTG-3′, reverse primer 5′-GGCACCAACACGTCCTCTCT-3′; aggrecan, forward primer 5′-AAATATCACTGAGGGTGAAGCCCG-3′, reverse primer 5′-ACTTCAGGGACAAACGTGAAAGGC-3′; GAPDH, forward primer 5′-ATTCTGGCAAAGTGGACATCGTCG-3′, reverse primer 5′-ATGGCCTTTCCATTGATGACGAGC-3′.

### Western blot analysis

The expression of CD44, ADAM10, and SOX9 protein was evaluated by Western blot using cell lysates. Cells cultured on silicone chambers were trypsinized and centrifuged first. Total protein was extracted from cell pellets with Cell Lysis Buffer (Cell Signaling, USA) containing a protease inhibitor cocktail. Samples were separated by 10% SDS-PAGE under reducing conditions and transferred to a nitrocellulose membrane. An antibody specific for the cytoplasmic tail of CD44 (anti-cytotail) was used to detect CD44-ICD^11,39^. Antibodies against ADAM10 (#14194), SOX9 (#14366), Myc (#2276), and beta-actin (#4970) were purchased from Cell Signaling (USA). Band intensities were captured with a digital image scanner and quantified using densitometry software (CS Analyzer 3.0; ATTO, Tokyo, Japan)^40^.

### Statistical analysis

Values are expressed as mean ± standard deviation (SD). The Kruskal Wallis test was used for multiple-group comparisons and the Holm post-hoc test was used to evaluate the significance of individual differences in two-group comparisons only when the Kruskal Wallis test indicated significance. P < 0.05 was considered statistically significant. All statistical analyses were carried out using EZR (Saitama Medical Center, Jichi Medical University, Saitama, Japan; http://www.jichi.ac.jp/saitama-sct/SaitamaHP.files/statmed.html)^41^.

## Supplementary information

Supplementary Information
