# Supplementary material for: Inhibition of CD44 intracellular domain production suppresses bovine articular chondrocyte de-differentiation induced by excessive mechanical stress loading
Source: Sci Rep. 2019 Oct 17;9:14901. doi: 10.1038/s41598-019-50166-4 (PMC6797729; doi:10.1038/s41598-019-50166-4)
Supplement: Supplementary file 1 — Supplementary Information [file 41598_2019_50166_MOESM1_ESM.docx]

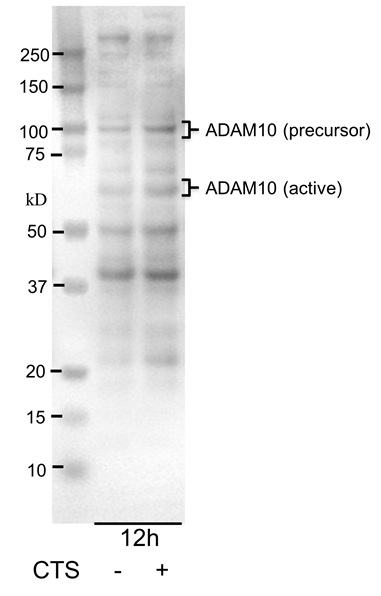


Supplemental figure 1. Entire blot of ADAM10 demonstrated in Figure 2C.

**
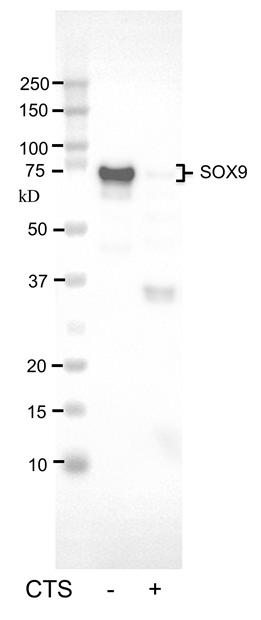
**

Supplemental figure 2. Entire blot of SOX9 demonstrated in Figure 3A.
